# Pirogoff’s Operation

**Published:** 1874-01

**Authors:** 


THE CLINIC:

PIROGOFF'S OPERATION—*Clinic of Prof. W. W. Dawson.*—Amputation at the ankle joint has been steadily growing in the confidence of the profession since it was so favorably presented and defended by Professor Syme, in 1842. Dr. Stephen Smith has shown that it is more than one-third less fatal than all the other amputations of the leg and foot.

The operation now known as "Syme's amputation" consists in the removal of the foot, the malleoli and the articular surface of the tibia. Pirogoff, in 1852, suggested a modification, which consists in leaving a portion of the os calcis in the posterior flap.

Admirable as are the stumps made by Syme's operation, they are surpassed in usefulness by those resulting from Pirogoff's modification. The limb is longer, the cushion more firm and resisting, enabling the patient to move about in the ordinary avocations of life with the most remarkable ease and facility.

Prof. Gross errs, certainly, when in speaking of the stump made by amputation at or near the ankle joint, he says: "It may answer very well when the patient is wealthy; but if he be poor, and obliged to work for his daily subsistence, he will generally get along much better with an artificial limb than with a stump that affords, even under the most propitious circumstances, only a miserable basis of support, and is besides constantly liable, from the slightest causes, to pain, irritation and ulceration."

In contrast with Gross' estimate of the value of such stumps, let me give you the opinion of Sir William Ferguson. "A long stump and a perfect covering to the end—a covering more perfect than that of any other stump; for the reason that every bit of soft material on which we naturally stand, is still preserved for the future basis of this support. * * * The soft tissue under the os calcis is the only part of the lower extremity which has been designed by nature for this purpose; and, with a happy idea Mr. Syme proposed to reserve this tissue."

The young man upon whom I propose to perform the Osteoplastic operation of Pirogoff, had his foot crushed three years ago. So anxious is the patient that a portion of his foot may be saved, that I elect to perform Pirogoff's modification rather than Syme's operation. Should I find, however, the os calcis diseased, the part of the os calcis left can be easily removed, the plantar flap shortened, and the amputation converted into Syme's.

The operation, though much slower than ordinary amputation, is nevertheless a very simple one. In making the plantar flap, I cut directly down to the bone, obliquing the incision from the front of the malleoli downwards and forwards, so that when it reaches the sole of the foot it shall be one inch in front of the place of beginning. The anterior flap is made with its convexity forwards. The division of the ligaments completes the disarticulation. I now apply the saw to the os calcis behind the astragalus, and cut in the direction of the first incision. The malleoli and articular surface are taken off together. The flap is now brought forward and the divided bones are placed in contact and retained in that position by the firm application of straps passing over the front and rear of the leg.

